# A national macroinvertebrate dataset collected for the biomonitoring of Ireland’s river network, 2007–2018

**DOI:** 10.1038/s41597-020-00618-8

**Published:** 2020-08-25

**Authors:** Hugh B. Feeley, Catherine Bradley, Gary Free, Bryan Kennedy, Ruth Little, Neasa McDonnell, Caroline Plant, Wayne Trodd, Caroline Wynne, Shane O’ Boyle

**Affiliations:** 1 Environmental Protection Agency, Richview, Clonskeagh Road, Dublin 14, D14 YR62 Ireland; 2 Environmental Protection Agency, John Moore Road, Castlebar, Co. Mayo, F23 KT91 Ireland; 3 Environmental Protection Agency, Inniscarra, Co. Cork, P31 VX59 Ireland; 4 Environmental Protection Agency, The Glen, Monaghan, H18 YT02 Ireland; 5 Present Address: Institute for Electromagnetic Sensing of the Environment, National Research Council, Milan, 20133 Italy

**Keywords:** Entomology, Freshwater ecology

## Abstract

The Environmental Protection Agency (EPA) in Ireland is responsible for the ecological monitoring and assessment of 37 hydrometric areas covering 46 river catchments and over 13,000 km of river channel nationwide. The national river monitoring program commenced in 1971 and has developed further since 2007 into the National Rivers Water Framework Directive (WFD) Monitoring Program following the implementation of the WFD across the European Union. The monitoring program is designed to obtain sufficiently representative information to assess ecological quality for each water body assessed. Consequently, macroinvertebrate data have been collected at over 2,900 river survey stations on a minimum 3-year cycle to fulfil these requirements. While the EPA has collected these data for water quality assessments we recognize that the data have value beyond this one purpose. We provide a summary of how these 10,987 data records, covering the years 2007 to 2018, have been collected and used to deepen understanding of water quality, biodiversity and general ecological health of Ireland’s river network.

## Abstract (summary)

**Table:** 

Measurement(s)	aquatic macroinvertebrate taxa • invertebrate
Technology Type(s)	Observation
Factor Type(s)	year of data collection
Sample Characteristic - Organism	aquatic macroinvertebrates
Sample Characteristic - Environment	watershed • river • environment associated with an aquatic invertebrate
Sample Characteristic - Location	Ireland

Machine-accessible metadata file describing the reported data: 10.6084/m9.figshare.12727631

## Background & Summary

Ireland (see usage notes) has over 84,800 km of river channel with streams, rivers and tributaries flowing from their headwaters to the sea through a vast network of channels. The national river monitoring program, established in Ireland in 1971 by An Foras Forbartha (succeeded by the Environmental Protection Agency (EPA) in 1993), is designed to provide representative information on the entire river network through the monitoring of discrete sections of river called water bodies. There are 3,192 river water bodies in Ireland and the national river monitoring program is designed to obtain sufficiently representative information to assign a status to each of these water bodies. All the major rivers and their significant tributaries are included in the monitoring program (https://gis.epa.ie/EPAMaps/) which has been carried out on a continuous basis since 1971 when 2,900 km of river channel was first surveyed. The surveys presently combine general chemical and biological assessments with approximately 13,200 km of river channel surveyed over a 3-year cycle^1–3^.

A summary of historical information on Irish water quality (covering rivers, lakes, ground, estuarine and coastal waters) is available in O’Boyle *et al*.^3^ and Trodd and O’Boyle^4^ (but see https://www.epa.ie/ for regular updates). In Ireland, the most commonly encountered forms of pollution in rivers are eutrophication and organic pollution^3,5,6^. Less frequently encountered are non-organic types of pollution such as toxic pollution (e.g. by sheep treatment or industrial chemicals)^3,7^, siltation (e.g. arising from cattle access over-grazing, drainage, quarrying or stone-cutting operations)^3,8,9^ and acidification in sensitive afforested areas^10^. Similarly, hydromorphological alteration to river channels can have influential and adverse effects on aquatic organisms^3^.

The Water Framework Directive (WFD), establishing a framework for European Union (EU) community action in the field of water policy with the objective to maintain or restore aquatic ecosystems, was adopted by the EU in October 2000 (2000/60/EC) and aims to achieve these objectives through a process of river basin management planning supported by status assessments derived from monitoring programs. As part of the WFD implementation process, the EPA was required to develop a new monitoring program for surface (rivers and lakes), ground, estuarine and coastal waters. The monitoring program became operational in December 2006 and in early 2007 the new WFD rivers monitoring program replaced the previous national river monitoring program^11^. The EPA is responsible for overseeing the WFD monitoring of biological elements, which in rivers focuses mainly on the assessment of aquatic macroinvertebrates to aid in the establishment of ecological status (see Methods for more information).

The data described here were originally collected for one purpose – to assess rivers nationwide to determine the quality of the macroinvertebrate communities across the country as part of the WFD ecological status assessment. These assessments indicate changes that pollution brings about in the benthic macroinvertebrate communities, i.e. larval insects (e.g. mayflies, stoneflies, caddisflies, beetles, etc.) together with crustaceans (e.g. shrimps), snails and bivalves, worms, and leeches. These changes reflect the varying sensitivities of the different groups of macroinvertebrates to the stresses caused by pollution, with sensitive species being progressively replaced by more tolerant forms as pollution increases. Such effects are well documented in the Irish and international literature (e.g. Kail *et al*.^12^).

While the EPA has collected this data for water quality assessments we recognize that the data have value beyond this one purpose. This dataset has the potential to be used to address many questions relating to our river environment including, for example, biodiversity assessments, species atlases and International Union for Conservation of Nature red listing, by providing an invaluable spatial and temporal dataset. Our purpose here is to widen the knowledge of, and accessibility of, these data. To date several projects and studies have acquired and utilized these data; for example, Donohue *et al*.^5^, Bennett *et al*.^13^, White *et al*.^14^, Feeley *et al*.^15,16^ and Kelly-Quinn *et al*.^7^, examining water quality and ecological relationships, ecology, biodiversity, species distributions and other work such as species red listing.

The biological program and macroinvertebrate data described here includes 2,976 unique biomonitoring stations and 10,987 individual surveys, assessed between 2007 and 2018, covering four 3-year reporting cycles.

## Methods

### Sampling rational and design

**Fig. 1:**
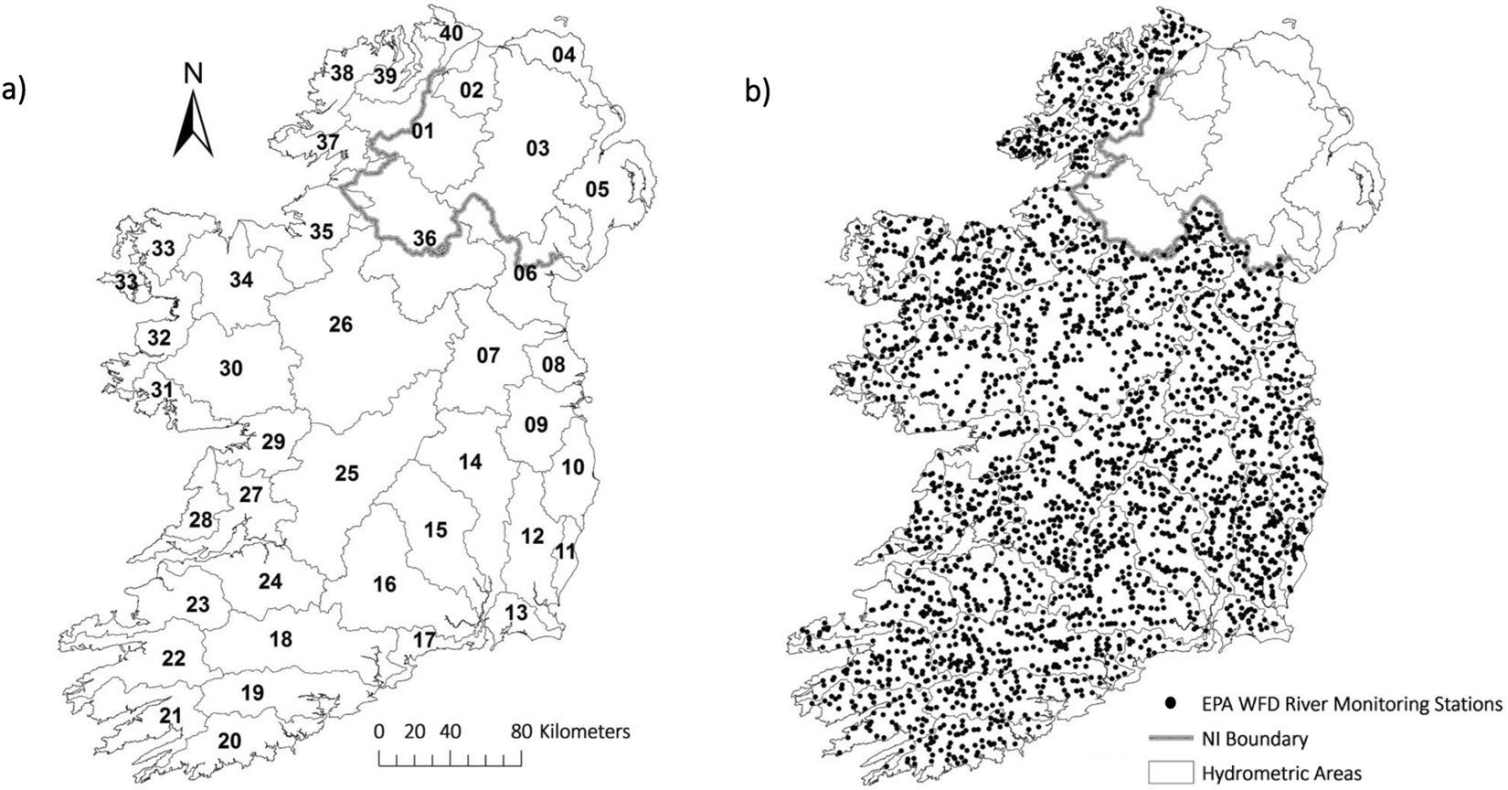
(**a**) Map of hydrometric areas (HA) in the dataset and (**b**) locations of all river biomonitoring stations 2007–2018 in Ireland. Both maps created using EPA data. See Table 5 for more details.

The EPA in conjunction with local authorities and other public bodies in Ireland has undertaken a substantial characterization of the physical water environment and the impact of human activities on waters^17^. Therefore, the monitored river water bodies in Ireland and the national river monitoring program are designed to obtain sufficiently representative information across river typologies and on significant pressures to assign a WFD status to each water body across our entire river network (Fig. 1).

The data collected covers the range of ecological conditions found in Irish rivers to assist the assignment of an ecological status as required by the WFD. Ecological status is an assessment of the quality of the structure and functioning of surface water ecosystems and it highlights the influence of pressures (e.g. pollution and habitat degradation) on several identifiable quality elements. As part of the WFD, ecological status is determined for each of the surface water body categories (i.e. rivers, lakes, transitional waters and coastal waters) using intercalibrated (see Technical Validation for further details) biological quality elements (BQEs) and supported by physico-chemical and hydromorphological quality elements. Ecological status for surface water bodies is primarily driven by the BQEs, namely fish, aquatic flora, macroinvertebrates and phytoplankton. The overall ecological status classification for any water body is determined, according to the ‘one out, all out’ principle, by the element with the worst status out of all the biological and supporting quality elements. In Ireland, macroinvertebrates are the main BQE determining the ecological status in rivers^3^.

The WFD requires BQE scores to be expressed as an Ecological Quality Ratio (EQR) to standardize and provide a common scale of ecological quality across participatory Member States using differing national methods^18^. The EQR is determined by expressing the observed result over the expected result which calculates a ratio score (Table 1). The ‘expected’ or ‘reference’ condition (EQR close to 1) is the natural, undisturbed environment, i.e. the benchmark. The assessment of the scale of anthropogenic pollution in any water body is based on the extent of deviation from expected reference conditions and follows the definitions as outlined in the WFD (Table 1). For example, ‘High status’ is defined as the biological, chemical and morphological conditions associated with no or very low human pressure, and therefore, little or no deviation from reference, ‘Good status’ means ‘slight’ deviation, ‘Moderate status’ means ‘moderate’ deviation, and so on. EQRs provide a common scale to ensure comparability across different pressures, allowing water managers to easily recognise and characterize impact facilitating the development of mitigation measures to restore or preserve ecological status^17^. To assess the network of rivers in Ireland, monitoring stations cover all 37 hydrometric areas (HA) providing a full national coverage (Fig. 1).

**Table 1:** The Q-value, ecological quality ratio (EQR)*, and corresponding WFD status and pollution gradient resulting from anthropogenic pressures.

Q-value Score	EQR	WFD Status	Pollution Gradient	WFD Color Code^†^
Q5	1.00	High	Unpolluted	Blue
Q4-5	0.90	High	Unpolluted	Blue
Q4	0.80	Good	Unpolluted	Green
Q3-4	0.70	Moderate	Slightly polluted	Yellow
Q3	0.60	Poor	Moderately polluted	Orange
Q2-3	0.50	Poor	Moderately polluted	Orange
Q2	0.40	Bad	Seriously polluted	Red
Q1-2	0.30	Bad	Seriously polluted	Red
Q1	0.20	Bad	Seriously polluted	Red

*Full details are available in Toner *et al*.^1^. ^†^Colors relate to the ecological status classification as outlined within the WFD^29^.

### Field sampling and data collection

River macroinvertebrates are collected from June to September each year, when flows are likely to be relatively low. Occasionally, for operational or weather-related reasons, surveys may occur outside of this period. Two approached are used. The first, and principal methodology used (96.7% of surveys in dataset), is by kick-sampling with a standard pond net (230 × 225 mm frame with 1 mm mesh). In this approach a semi-quantitative two-minute macroinvertebrate kick-sample is collected from the riverbed preferably from the faster flowing riffle habitats^19^. A further one-minute hand search is carried out to locate macroinvertebrates that remain attached to the underside of the cobbles^19^. Depending upon the proportion of various habitats (e.g. glides, margins, pools), time may also be spent sampling these habitats with operators moving location approximately every 4 to 5-seconds over a 50 m stretch. Similar studies in Ireland and elsewhere have found that this sampling approach is sufficient to achieve a suitable representation of taxa for bioassessment of lotic habitats^20,21^. Occasionally, when the substratum (e.g. bedrock) or flow conditions make kick-sampling difficult, or the abundance of macroinvertebrates collected is extremely low (e.g. toxic pollution, see Kelly-Quinn *et al*.^7^), it may have been necessary to spend a longer amount of time sampling the river to accumulate a sufficient diversity and abundance of macroinvertebrates. In fast flowing steep rivers, it may have been necessary to kick deeper into the riverbed to disturb the organisms and include more of the marginal areas to ensure taxa are recorded^19^. This sampling approach requires avoidance of obvious localized disturbance (e.g. cattle access points) which may adversely influence the sample taken.

If the river depth is too deep to wade, a separate approach is taken. In this scenario, a bankside extension net sampling approach for deep (non-wadable) rivers is used to collect macroinvertebrates. It must be noted that this methodology is used less frequently than the kick-sampling approach. If employed, the depth and number of extension poles attached to a modified hand net will vary on a site by site basis. The net (frame and mesh dimensions as above) is then pulled upstream along the riverbed, generally at a perpendicular angle to the bank to cover as much surface area as possible with operators moving location after every pull over a 20 to 50 m stretch. The net may also need to be emptied between pulls to ensure that macroinvertebrates already collected are not lost inadvertently during the next pull. The extension net is also used to sweep along the water surface and marginal vegetation. This approach is conducted for a minimum of five minutes or until a representative sample is obtained (see Technical Validation for more details).

Once a live sample is collected it is assessed on the riverbank and the EPA Q-value classification is assigned (see Toner *et al*.^1^ for more details). This involves recording the taxa present at a suitable and attainable (under field conditions) taxonomic resolution (Table 2) and their categorical relative abundance (Table 3), determined using approximate counts. Once all taxa and their relative abundance have been recorded, the sample is returned to the river. Potential users should note that actual numbers of taxa have not been recorded and are therefore unavailable within the dataset. Similarly, taxonomic resolution may vary from what is outlined in Table 2. Indeterminate specimens may be brought back to the laboratory for identification under a microscope. Taxa are also occasionally returned to the laboratory and identified by microscope as a quality control measure. A brief description of the Q-value ecological quality rating (EQR) is outlined in Table 1. The typology of each river station is described in Table 4, after Kelly-Quinn *et al*.^22,23^.

**Table 2:** The level of macroinvertebrate identification carried out in the field during WFD biomonitoring assessments.

Group	Level of Identification
Platyhelminthes	Genus
Oligochaeta	Family
Hirudinea	Genus
Gastropoda	Genus
Bivalvia	Genus
Crustacea	Family/genus
Plecoptera	Genus
Ephemeroptera	Genus, except *Baetis rhodani*/*atlanticus*
Trichoptera	Genus
Odonata	Genus
Megaloptera	Genus
Hemiptera	Genus
Coleoptera	Family
Diptera	Family
Chironomidae	*Chironomus* spp. or non-*Chironomus* spp.
Acari	Subclass

Note: resolution may vary within each survey year and survey station.

**Table 3:** Abundance categories for macroinvertebrates recorded in the field during WFD biomonitoring assessments.

Abundance category	Relative Percentage Frequency of Occurrence
Single	—
Few	1–5%
Common	6–20%
Numerous	21–50%
Dominant	51–74%
Excessive	>75%

**Table 4:** Typologies of Irish rivers.

Code	Catchment Geology*	Description	Hardness
1	100% Siliceous	Soft water	<35 mg CaCO_3_/l
2	1–25% Calcareous^†^	Medium hardness	35–100 mg CaCO_3_/l
3	>25% Calcareous	Hard water	>100 mg CaCO_3_/l
**Code**	**Slope (m/m)**	**Description**	
1	≤0.005	Low Slope	
2	0.005–0.02	Medium Slope	
3	0.02–0.04	High Slope	
4	>0.04	Very High Slope	

Example of Type Codes: The two codes from above are combined in order geology first digit and slope second digit e.g. A code of 31 indicates a calcareous low-slope site. For more information see Kelly-Quinn *et al*.^22,23^, and The Characterisation and Analysis of Ireland’s River Basin Districts. National Summary Report^30^. * % bedrock in upstream catchment by type. ^†^Mixed geology.

Each hydrometric area (Table 5 and Fig. 1) is generally surveyed on a three-year cycle; however, full surveys of certain hydrometric areas may be spilt across two concurrent years (e.g. HA 25), and on occasion a subset of stations were surveyed/resurveyed outside of the main survey year to closely track any progress in status changes following the implementation of a program of measures (Table 6). Certain stations were sampled on a more frequent basis such as seriously polluted sites (i.e. Red dot sites – Fanning *et al*.^24^), WFD high status objective sites, priority areas for action identified in Ireland’s national river basin plan^17^ and occasional sites of interest to local authorities and the EPA Office of Environmental Enforcement.

**Table 5:** Hydrometric area (HA) codes and HA names on the island of Ireland.

HA	Name	HA	Name
01^†^	Foyle	21	Dunmanus-Bantry-Kenmare
02*	Lough Foyle	22	Laune-Maine-Dingle Bay
03^†^	Lough Neagh & Lower Bann	23	Tralee Bay-Feale
04*	Bush & North East Coast	24	Shannon Estuary South
05*	Belfast Lough & East Down	25	Lower Shannon
06^†^	Newry, Fane, Glyde and Dee	26	Upper Shannon
07	Boyne	27	Shannon Estuary North
08	Nanny-Delvin	28	Mal Bay
09	Liffey and Dublin Bay	29	Galway Bay South East
10	Ovoca-Vartry	30	Corrib
11	Owenavorragh	31	Galway Bay North
12	Slaney & Wexford Harbour	32	Erriff-Clew Bay
13	Ballyteigue-Bannow	33	Blacksod-Broadhaven
14	Barrow	34	Moy & Killala Bay
15	Nore	35†	Sligo Bay & Drowse
16	Suir	36†	Erne
17	Colligan-Mahon	37	Donegal Bay North
18	Blackwater (Munster)	38	Gweebarra-Sheephaven
19	Lee, Cork Harbour and Youghal Bay	39	Lough Swilly
20	Bandon-Ilen	40	Donagh-Moville

*No coverage of HA 02, 04, 05 which are in Northern Ireland (United Kingdom). ^†^Only covers HA 01, 03, 06, 35, 36 in the Republic of Ireland. See Fig. 1.

**Table 6:** The number of river biomonitoring stations assessed by year and hydrometric area (HA), held by the EPA* 2007 to 2018.

HA	2007	2008	2009	2010	2011	2012	2013	2014	2015	2016	2017	2018	Total
**01**	13	3	—	—	36	—	41	4	—	42	1	1	**141**
**03**	15	—	—	19	—	—	19	—	—	—	19	1	**73**
**06**	2	2	44	1	12	45	2	—	50	—	—	63	**221**
**07**	1	1	79	—	—	100	—	5	89	—	—	92	**367**
**08**	1	26	1	26	—	—	—	28	—	—	27	5	**114**
**09**	64	2	—	71	—	—	68	—	1	69	1	5	**281**
**10**	1	1	59	1	1	68	—	—	70	—	—	66	**267**
**11**	20	1	1	19	—	—	24	—	1	—	24	1	**91**
**12**	96	—	—	111	—	—	115	—	—	115	—	4	**441**
**13**	18	—	—	21	—	2	22	—	—	—	22	—	**85**
**14**	5	2	109	—	134	—	—	89	36	1	105	30	**511**
**15**	95	2	—	84	12	—	94	—	—	97	1	7	**392**
**16**	—	126	3	—	127	13	1	138	—	—	138	4	**550**
**17**	16	—	—	17	—	—	17	—	—	16	—	1	**67**
**18**	1	1	142	—	1	139	—	—	162	—	—	160	**606**
**19**	—	69	—	—	75	—	—	77	1	—	78	10	**310**
**20**	—	—	59	—	—	59	—	—	64	—	—	64	**246**
**21**	—	—	54	—	—	54	—	—	59	—	—	58	**225**
**22**	76	—	—	30	30	21	76	—	9	83	—	13	**338**
**23**	55	1	1	—	61	—	—	56	—	—	57	6	**237**
**24**	—	63	—	—	45	19	—	65	—	—	66	8	**266**
**25**	3	138	54	1	111	128	—	158	73	—	160	86	**912**
**26**	1	178	19	—	211	2	2	215	2	1	212	5	**848**
**27**	59	—	—	31	42	—	71	9	—	79	—	1	**292**
**28**	1	—	34	—	—	38	—	—	37	—	—	36	**146**
**29**	—	1	28	—	—	28	—	—	30	—	—	30	**117**
**30**	—	1	87	—	—	82	—	—	89	1	1	93	**354**
**31**	—	—	19	—	—	20	—	—	19	—	—	19	**77**
**32**	—	3	53	—	3	56	—	60	—	—	59	11	**245**
**33**	—	38	—	—	24	15	—	41	—	—	41	2	**161**
**34**	101	2	2	108	21	—	106	2	22	132	—	12	**508**
**35**	—	—	70	—	—	66	6	—	71	—	—	71	**284**
**36**	84	—	7	106	1	8	107	2	8	—	107	12	**442**
**37**	—	42	—	13	22	—	—	—	62	—	—	62	**201**
**38**	3	—	59	—	—	66	2	—	66	—	—	66	**262**
**39**	39	—	—	51	1	—	53	1	—	52	—	3	**200**
**40**	15			27	1		27	1	1	27		10	**109**
**Total**	**785**	**703**	**984**	**737**	**971**	**1029**	**853**	**951**	**1022**	**715**	**1119**	**1118**	**10987**

*No coverage of HA 02, 04, 05 which are in Northern Ireland and only coverage of HA 01, 03, 06, 35, 36 in the Republic of Ireland.

Within each hydrometric area, water bodies may have one or more sampling stations along their continuum. The number of stations may also vary between survey years, although, unless health and safety, or other unforeseen circumstances arise, the EPA attempt to sample the same stations in each survey cycle. Similarly, the numbers of water bodies and stations sampled within each hydrometric area will reflect the geographical area and length of river network.

## Data Records

Although the EPA has collected river macroinvertebrate data in Ireland since 1971, only data from 2007 onwards is available digitally at present. While efforts are being made to digitize pre-2007 data, the current paper and associated dataset only relates to the period 2007 to 2018 covering four full WFD river invertebrate biomonitoring cycles. Table 6 highlights the availability of data in each HA over the data collection period. The dataset of macroinvertebrate taxa includes several descriptive and complimentary fields for each record (Table 7).

**Table 7:** Dataset fields descriptors and explanatory notes.

Field descriptor	Explainer
HA	Hydrometric Area, format: e.g. 25 [=Lower Shannon (see Table 5)]
WFD River Waterbody Code	Unique river waterbody identifier code
WFD River Waterbody Name	Unique river waterbody identifier name
River Name	River name
Station Code	Unique WFD station code
Station Name	Unique WFD station name
Local Authority	Local government administrative body
Altitude (m)	Altitude, format: meters (above sea-level)
Station Typology	Typology of station, if available. See Table 4 for descriptions
Longitude	Geospatial location of station
Latitude	Geospatial location of station
Channel Description	Predominant channel form sampled, if known
Recent Flood	At time of survey: ‘Yes’ or ‘No’, if known
Q-Value Number	Station Q-value score (see Table 1)
Kick Sample (mins)	Duration of kick sampling, if utilized, format: minutes
Stone Wash Sample (mins)	Duration of stone washing, if utilized, format: minutes
Bankside Extension Pole Sample (mins)	Duration of bankside extension pole sampling, if utilized, format: minutes
Field Sheet Date	Date of survey, format: DD/MM/YY
Year	Year of survey, format: YYYY

The complete list of taxa included in the dataset is fixed but note that because taxonomic resolution varies annually and between stations some minor editing may be required before usage. The associated relative abundance categories are outlined in Table 3. Note if a taxon has no relative abundance it was not recorded. Nomenclature is based on that supplied by the British Natural History Museum (https://www.nhm.ac.uk/our-science/data/uk-species/species/index.html [accessed 01 July 2020]) and Freshwater Animal Diversity Assessment (FADA) Project (http://fada.biodiversity.be/).

The dataset includes 10,987 records (see Table 6), each representing an associated list of macroinvertebrate taxa from a unique date and monitoring station. All data collections occurred between 17 April 2007 and 18 October 2018 and are available in figshare^25^.

## Technical Validation

The data described here were collected using the EPA Q-value classification as described briefly above with full details available in Toner *et al*.^1^. This approach, established and developed by An Foras Forbartha, was tested and intercalibrated by the EPA against other European macroinvertebrate assessment methods (2008/915/EC)^26^ and was adopted as the official macroinvertebrate classification system for assessing rivers in Ireland (Statutory Instrument No. 272 of 2009). More detailed information on intercalibration of this metric is available in McGarrigle and Lucey^27^ and a comparison with other intercalibrated European macroinvertebrate metrics is available in Bennett *et al*.^13^. Note that the Q-value is based on the well-established sensitivities, abundance and diversity of macroinvertebrates and their relationship to water quality. The system is considered a proprietary expert system and is generally only applied from June through September.

In terms of data collection, operator harmonization occurs annually at the beginning of each sampling season to ensure all operators in the field use identical and replicable approaches to sample collection, sample size, macroinvertebrate identification and data recording. All data collected is checked and accurately transferred to the in-house database. All data are double checked by an independent user to ensure all data collected have been accurately transcribed.

**Fig. 2:**
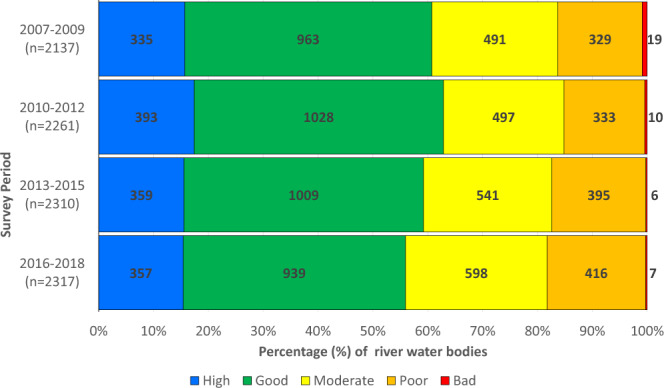
National trends in macroinvertebrate quality of water bodies using the Q-value rating system calculated using the data collected by the EPA between 2007 and 2018. Number in parentheses on y-axis is total number of water bodies assessed. The color legend reflects ecological conditions as described in Table 1. Adapted from O’Boyle *et al*.^3^.

The long-term collection of this data and the harmonization approaches employed allow the production of a time series which provides a valuable record of environmental change in Irish rivers at a national level (Fig. 2). Additionally, Donohue *et al*.^5^ found highly significant relationships between Q-values and measures of urbanization and agricultural intensity, densities of humans and cattle within a catchment, but also chemical indicators of water quality, namely molybdate-reactive phosphorus, ammonia, total oxidized nitrogen, biological oxygen demand, pH and conductivity. There is also evidence to show macroinvertebrate data collected for Q-value determination has a relationship with fish diversity and density^28^.

## Usage Notes

The use of the terms ‘Ireland’, ‘Republic of Ireland’ and ‘Irish’ are used interchangeably throughout this paper and refer only to the State known officially as ‘Ireland’ and described as the ‘Republic of Ireland’, and does not include the entirety of the island of Ireland, which includes ‘Northern Ireland’, part of the United Kingdom.

Data that is produced directly by the EPA is free for use; however, where data are used in publications and/or presentations, in full or partial, the EPA should be acknowledged. An example of how to acknowledge the use of EPA data is, but not limited to, the following: ‘Data included [in this study/review/other] was provided by the Environmental Protection Agency (Ireland)’.

The EPA take no responsibility for any third-party use or analysis of data, nor does it endorse any third-party opinions or conclusions reached using this data. We also ask that users notify the authors of any errors or omissions identified in the data so that we can correct same. Note that the most recent and historic biological survey summaries for rivers are freely available at http://www.epa.ie/QValue/webusers/ and mapped at https://gis.epa.ie/EPAMaps/. The data presented here underpins these survey results for the period 2007 to 2018. Additional water body information, including some physical characteristics (e.g. water body length) and chemistry (where measured) are available at https://www.catchments.ie/data/.
